# The Patient Activation through Community Empowerment/Engagement for Diabetes Management (PACE-D) protocol: a non-randomised controlled trial of personalised care and support planning for persons living with diabetes

**DOI:** 10.1186/s12875-020-01173-2

**Published:** 2020-06-19

**Authors:** Wee Hian Tan, Victor Weng Keong Loh, Kavita Venkataraman, Shoon Thai Choong, Yii Jen Lew, Meena Sundram, Keith Tsou, Soon Guan Tan, Brent Gibbons, Vikki Entwistle, Doris Young, E Shyong Tai, Tong Wei Yew

**Affiliations:** 1 National University Polyclinics, National University Health System, Singapore, Singapore; 2 Division of Family Medicine, Yong Loo Lin School of Medicine, National University of Singapore, Singapore, Singapore; 3 Saw Swee Hock School of Public Health, National University of Singapore, Singapore, Singapore; 4 Centre for Biomedical Ethics, Yong Loo Lin School of Medicine, National University of Singapore, Singapore, Singapore; 5 Division of Endocrinology, University Medicine Cluster, National University Health System, Singapore, Singapore

**Keywords:** Care and support planning, *Patient activation.*, *Self-management.*, *Diabetes mellitus.*, *Long term conditions.*, *Primary care.*

## Abstract

**Background:**

Personalised care and support planning (CSP) has been shown to improve diabetes outcomes, patient experience and provider morale in the care of persons living with diabetes. However, this has not been confirmed in controlled studies. Patient Activation through Community Empowerment/Engagement for Diabetes Management (PACE-D) is a pragmatic controlled trial that evaluates the effectiveness of personalised CSP in persons living with diabetes in the public primary care setting in Singapore.

**Methods:**

Teamlet-empanelled patients with diabetes at four polyclinics are recruited for this study. Participants who attend either of the two Intervention clinics are sent their investigation results in a care planning letter (CPL) to *prepare* them for the CSP conversation. This conversation is facilitated by a trained CSP practitioner who engages them in *discussion* of concerns, goals and action plans, and *documents* their plans for subsequent *review*. Participants in the two Control clinics will receive standard diabetes care. Participants will complete two or more CSP conversations (Intervention) or regular consultations (Control) at the annual review visits within the 18 months of the study. The sample size is calculated at 1620 participants, with glycated haemoglobin (HbA1c) as the primary outcome measure. Secondary outcome measures include patient activation (as measured by PAM-13) and changes in healthcare utilisation and cost.

**Discussion:**

This study is a pragmatic trial that evaluates the effectiveness of personalised CSP in persons living with diabetes in a real world setting. It promises to provide insights with regard to the implementation of this model of care in Singapore and the region.

**Trial registration:**

ClinicalTrials.gov Identifier NCT04288362. Retrospectively registered on 28 February 2020.

## Background

With more than 60% of the 422 million persons living with diabetes mellitus, Asia is poised to be the global epicentre of this long term condition (LTC) in this century [1, 2]. Inhabited by 5.7 million residents and nestled within Southeast Asia, the multicultural city-state of Singapore is a microcosm of the sociocultural norms of the region. Given a prevalence of 11.3% among adults in 2010, Singapore has one of the highest rates of diabetes worldwide [3, 4]. Current estimates predict that one million residents will have diabetes in 2050 compared to 440,000 in 2014 [5, 6]. Healthcare cost-wise, USD 1.9 billion will be spent on diabetes in 2050 compared to USD 790 million in 2010 [7]. In response to these projections, Singapore declared a nationwide, long-term “war on diabetes” in 2016 [8]. Support for active self-management of persons living with diabetes was identified as a key focus of the campaign [5].

Over the past decade, it has been increasingly recognised that outcomes for persons living with diabetes and for the healthcare system, are better when persons with LTCs are empowered to take charge of their condition [9, 10]. In Singapore, however, the public primary care clinics (polyclinics) have been designed mainly for efficient medical problem solving rather than for systematic engagement of patients in long-term self-care. As an illustration, a typical consultation for an LTC at the polyclinic lasts 8–12 min, usually used to address new complaints, explain investigation results, problem-solve, provide patient education, and plan subsequent steps. The presence of comorbidities compounds the time-pressure. Most patients and doctors would find it difficult to consider the significance of adopting any positive health behaviour within the time constraints. Given that the bulk of patients with LTCs including diabetes are managed at the polyclinic, there is value in learning and adapting from best practices elsewhere [4].

### Personalised care planning

Persons with LTCs have important roles in managing their own health in the context of their daily lives, but often need support, including to develop the confidence and skills to adhere to medications, adopt and maintain healthy lifestyles, and know when and how to seek medical advice. Personalised care and support planning (CSP) has been proposed as a means to provide support from healthcare providers (HCPs) that is individualised to the needs of specific persons with LTCs and oriented to enable them not just to manage their LTCs well in biomedical terms but more broadly to live well with those LTCs [11, 12]. Personalised CSP entails a conversation or a series of conversations between the patient and the HCP when they jointly agree on goals and actions for managing the patient’s health problems [11, 12]. Evidence shows that personalised care planning, most effective when integrated in routine clinical practice, leads to improvements in certain indicators of physical and psychological health status of patients, and their capability to self-manage their condition as compared to usual care [11, 12].

### The care and support planning model

Year of Care Partnerships (YoC) in the United Kingdom (UK) has implemented and made iterative changes in the use of CSP for over a decade [11, 12]. The personalised CSP has been conceptualised as a meaningful conversation between partners that occurs within a “house of care” [13] with four components: The prepared person living with diabetes and the trained CSP practitioner form each of two walls of the house, the organisational processes that operationalise the CSP form the roof, while institutional support forms its foundation.

YoC’s experience over the past decade has shown that personalised CSPs have resulted in positive outcomes for patients, HCPs and healthcare organisations. Patients experience improved clinical outcomes in terms of glycated haemoglobin (HbA1c) and blood pressure within 3–5 years of enrolment, in parallel with increased engagement with positive health behaviours. Explicit orientation of care to support personal goals in CSPs was observed to raise practitioner morale and strengthen the practitioner-patient relationship; ripple-effects have been seen at the organisational level with improved levels of productivity at zero additional cost [7, 13]. As a further badge of its success, CSP practice has spread to multiple sites across England and Scotland, [11] and CSPs have been adopted as part of the core curriculum in General Practice training in the Royal College of General Practitioners (RCGP) since 2019 [14].

In Patient Activation through Community Empowerment/Engagement for Diabetes Management (PACE-D), the model of care from YoC has been adapted for the Singapore polyclinic context. Structured on the blueprint of the chronic disease model, [15] it places self-management at the forefront of diabetes management. The programme builds on the clinical experience in the management of chronic diseases in the polyclinic setting and on YoC’s extensive experience with the use of care plans. It is underpinned by the conceptual frameworks of the patient-centred consultation, the theories of adult education and self-efficacy, the concepts of self-management and self-empowerment, [9, 16–21] and has been described in terms of six programme theories in a recent realist evaluation [22].

This paper describes the design and significance of PACE-D, a pragmatic controlled trial that evaluates the effectiveness of personalised CSP in persons living with diabetes in the public primary care setting in Singapore.

### Primary objective

The primary objective of this study is to examine the effects of personalised CSP (Intervention) compared to the standard model of care (Control) on glycaemic control, as measured by HbA1c.

### Secondary objectives

The secondary objectives include investigating the change in patient activation [23], measured by Patient Activation Measure-13 (PAM-13), and the difference in healthcare utilisation and cost between the Intervention and Control.

The selection of the primary and secondary objectives reflects strong health policy interests in Singapore. The intervention designers and research team are aware that they can be in some tension with the idea that CSP involves working responsively with each patient to help them manage their life with the condition rather than (more narrowly) to manage the biomedical aspects of the condition well. To elucidate this further, the qualitative experiences of the participants and HCPs will be researched in parallel with this study.

## Methods/design

**Fig. 1:**
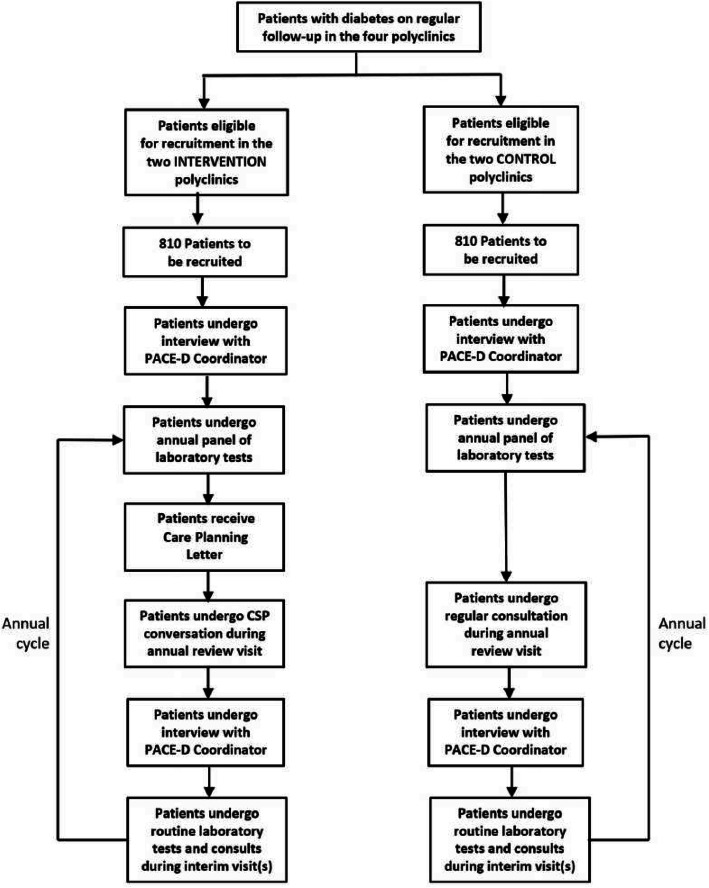
Participant flow in the Patient Activation through Community Empowerment/Engagement for Diabetes Management (PACE-D) study

PACE-D is a pragmatic non-randomised controlled trial that evaluates the effects of personalised CSP on persons living with diabetes over two annual reviews with two groups: Intervention experiences personalised CSP; and Control continues with the current care model. Figure 1 illustrates the flow diagram for study participants.

### Trial oversight

The PACE-D research committee meets quarterly to provide research oversight. Process measures are reported six-monthly to the Ministry of Health (MOH) that funds the project.

### Patient involvement

Feedback from patients recruited in two earlier pilots informed the design of the care planning letter, the length of the questionnaires, and the feasibility of the operational procedures.

### Study setting

PACE-D is conducted in the polyclinics at National University Polyclinics (NUP), the westernmost of three major administrative primary care healthcare groups in Singapore. Each polyclinic is staffed by 15–25 primary care physicians (PCPs) deployed in multi-disciplinary teams which include nurses and clinical pharmacists, with access to the psychologist, dietitian and medical social worker. In addition to the management of acute episodic conditions, the polyclinics provide for the efficient care of the patient with LTCs: laboratory services allow for rapid investigative reporting; surveillance programmes ensure the systematic detection of diabetic retinopathy, neuropathy and nephropathy; and workflows designed to target specific needs through the use of teamlets, [24] tele-consults, [25] and multidisciplinary case discussions [26, 27].

At the polyclinics under NUP, primary care is delivered to patients with LTCs through care teams known as teamlets. Each teamlet provides LTC care to a regular pool of approximately 5000 patients. It is formed by two PCPs and one care manager (CM) who is a chronic disease management-trained senior nurse. These three HCPs are supported by a care coordinator (CC) who schedules consultation and preventive health screening appointments for teamlet-empanelled patients.

In PACE-D, four of the six NUP polyclinics are involved in the study, with two of them designated as Intervention and the other two as Control. In each of these polyclinics, one teamlet is involved in the study and is supported by a PACE-D Coordinator (PC). The role of the PC is to recruit participants and obtain consent for participation, prepare the care planning letter (CPL) for mailing to the participants in the Intervention, and to administer questionnaires to all participants.

### Study population

The study population comprises patients currently receiving care from a designated teamlet in each of the four NUP polyclinics. Patients are recruited if they meet all of the following inclusion criteria: (1) adults with diabetes mellitus aged 21 years old and above; the (2) ability to provide informed consent; (3) ability to communicate in English, Malay or Chinese and (4) ability to comprehend the CPL on their own or with the assistance of family members.

Patients are not eligible for the study if they are (1) pregnant, or unable to engage with the CPL and/or CSP conversation because of any of the following: (2) cognitive impairment; (3) psychotic disorders; and (4) severe hearing/visual impairment.

### Study recruitment process

Each site has a PC who identifies eligible patients in the week’s appointment register. Potential participants are approached to participate in the study by the PC while they are in the waiting area prior to the consult, or by the HCP during the consultation. Consent is obtained from the patients by the PC if they are agreeable to participate. All enrolled patients in both Intervention and Control are each given an SGD $10 voucher by the PC after the completion of every CSP conversation (Intervention) or regular consultation (Control) at the annual review visit during the study.

## Trial intervention

### Current model for Diabetes care

At NUP polyclinics, patients with diabetes undergo an annual panel of laboratory tests in accordance with local clinical guidelines [28]. This includes the HbA1c, fasting capillary glucose, lipid profile, renal function, and urine albumin:creatinine ratio. The patient attends an annual review consultation in the following week with his/her teamlet HCP who jointly reviews the patient and laboratory test results. Depending on the patient’s condition and its control, he/she will be scheduled to attend one or more interim reviews (with laboratory tests performed as indicated) before the following annual review consultation.

### Intervention

In the PACE-D trial, patients enrolled in Intervention undergo personalised CSP. When it works as intended, it entails: [11].

1. *Preparation* – Two weeks before the scheduled appointment, the patient receives a CPL (Additional file 1) containing his/her most recent laboratory test results and condition-specific information, which act as reflective and agenda-setting prompts to prepare them for the upcoming CSP conversation.
2. *Conversation* – The prepared patient brings the expertise of his/her lived experience to meet the CSP practitioner who is the medical expert and trained in active listening and communication skills. The quality conversation facilitates goal setting and action planning in a meaningful way to the patient.
3. *Recording –* The CSP practitioner documents a summary of the points discussed and actionable plans in the electronic medical record (EMR). The patient is encouraged to write these plans directly on the CPL.
4. *Actions* – The patient commits to decisions and activities that facilitate self-management through non-traditional, formal and informal support from groups and peers. The PC may be involved in referring and coordinating these efforts.
5. *Review* – The patient attends an interim consult on a date fixed to review his/her LTCs and the progress of plans made.

### Control

Patients in Control will continue with the current care model. That means they will not receive the CPL. They will not participate in the CSP conversation but will continue to undergo their regular annual review consultation instead. However, during the consultation, they will receive a generic brochure that provides information about the available community programmes (common to both the Intervention and the Control) to support self-management. These patients will have to proactively sign up for these programmes.

### Training and support for the healthcare providers

All designated CSP practitioners attended a two-day PACE-D training workshop. The programme was facilitated by YoC-accredited trainers using training materials adapted from the YoC for the Singapore polyclinic context. The planned curriculum covered topics that ranged from the rationale and paradigms for CSPs, the processes involved, to hands-on skills-training for the conduct of CSP conversations. Content delivery occurred through a mix of presentations, role-plays, discussions and active reflection. This was followed by an additional CSP role-play practice session before they started CSP conversations with actual study participants. In all, there are 14 trained PACE-D CSP practitioners in NUP. Ongoing quarterly huddles are carried out by the trainers with these CSP practitioners to support fidelity to the intervention.

## Data collection

### Baseline data and questionnaire collection

The data collection points in PACE-D are summarised in Table 1. Upon enrolment, the PC administers a set of baseline questionnaires to capture details of socio-demographic profile (religion, current marital status, housing type, date of birth, ethnicity, gender, residential status, educational qualification), existing comorbidities (hypertension, hyperlipidaemia, ischemic heart disease, stroke, major depression, mood disorder of depressed type), anthropometric measurements (height and weight), healthcare utilisation in the past 1 year (for hospitalisation, emergency department visit, specialist outpatient clinic and the primary diagnosis of each visit), diabetes medication status (on anti-diabetes oral medication, on insulin therapy), lipid profile targets, [29] attendance at scheduled foot and eye screening in the past year, and self-care activities in the past year including smoking status, physical activity participation and community resource utilisation.

**Table 1:** Data collection points at baseline, first annual review and second annual review for participants

Study overview					
	Recruitment	Annual Review (AR) 1			AR2
**Time point**	*−3 Month*	*− 3 Week*	*−1 Week*	*Year 0*	*Year 1*
	*Enrolment Visit*	*Annual Blood Panel*	*Care Planning Letter*	*Annual Review Visit*	*AR2 will be same as AR1 in sequence*^*a*^
**Enrolment**:					
Eligibility Screen & Informed Consent	✓				
**Study groups**:					
Control		✓		*Standard Care*	Y
Intervention		✓	✓	*CSP*	Y
**Variables collected**:					
**Primary outcome**					
*HbA1c*		✓			Y
**Secondary outcomes**					
*PAM-13*	✓			✓	Y
*Healthcare Utilisation Survey*	✓			✓	Y
*Healthcare Utilisation, Cost Data*	*Extracted from medical records, billing information, ePOS, Oracle BI*				
**Other pre-specified outcomes**					
*Body Weight and Blood Pressure*		✓		✓	Y
*LDL-cholesterol*		✓			Y
*Exercise, smoking status, community resource utilisation*	✓			✓	Y
*Foot and Eye Screening*	✓			✓	Y
*Survey on perception of the new care model (Intervention only)*				✓	Y

✓ *– Performed, collected, administered through survey (whichever applicable)*

Y *– Collected or performed in the same order as the various time points and sections detailed under Annual Review 1*

^*a*^*Patient will be returning for their annual blood panel test prior to the annual review. Patients in the Intervention will receive their care planning letter. Following that, the patients will go for the annual review where they receive either the standard care (Control) or CSP (Intervention)*

PAM-13 is administered on this first visit to determine baseline patient activation levels in all patients. The PAM-13 is a short form comprising 13 item measures that ascertain self-reported knowledge, skill and confidence in the self-management of the individual’s own health or LTC. Patient activation is scored in the range of 0–100 and categorised into 4 levels, with higher scores being reflective of greater levels of activation and greater capacity for self-management [23].

### Clinical data collection and follow-up assessments

Clinical outcomes data used for assessment is collected as part of routine practice for diabetes management in NUP, based on local clinical practice guidelines, and will be extracted from the EMR for purposes of this study. The clinical measurements are as listed in Table 1.

Survey questionnaires administered following each of the scheduled annual reviews include PAM-13, engagement in self-care activities, diabetes medication status (binary question on oral anti-diabetic medication and insulin therapy), and foot and eye screening attendance. Patients in the Intervention will receive an additional survey to rate their perception of the new care model. Results of routine blood tests, clinical markers and survey questionnaires administered throughout the study will be stored into REDCap, a secure browser-based platform for capturing of consent forms and research data electronically.

### Healthcare cost and utilisation

Data related to healthcare cost and utilisation, specifically on the following, will be extracted by the National University Health System (NUHS) Academics Informatics Office.

- Healthcare utilisation in hospitals, including visits to emergency departments and hospitalisations, and their corresponding primary diagnosis;
- Number of visits to the specialist outpatient clinics (SOCs) and the names of the specialities;
- Number of visits to the polyclinics and their corresponding primary diagnosis;
- Total bill sizes, drug costs and bill breakdowns for the visits to emergency departments, hospitalisations, SOCs and polyclinics.

Data extraction from the Electronic Polyclinic Outpatient System (ePOS) and Oracle Business Intelligence (BI) platforms will be performed for the institutions under the purview of NUHS. These institutions include Alexandra Hospital, Ng Teng Fong General Hospital and National University Hospital.

## Outcome measures

### Primary outcome

- Change in HbA1c levels in patients receiving the CSP intervention compared with patients receiving standard care from baseline to the study endpoint as defined by the second annual review

The latest HbA1c reading prior to the first annual review will be taken as the baseline and the HbA1c on the second annual review will be taken as the endpoint. The baseline and endpoint for the other variables will be selected on a similar basis.

### Secondary outcomes

Secondary outcomes will be compared between patients receiving the CSP intervention with patients receiving standard care, from baseline to study endpoint.

#### Patient activation

- Change in mean PAM-13 score (continuous, 0 to 100) and patient activation levels (categorical, level 1 to 4)

#### Healthcare Services Utilisation and Cost

- Change in healthcare utilisation in terms of number of polyclinic, emergency department, hospital admissions and SOC visits from one year preceding recruitment to the period between the first and the second annual review
- Change in healthcare cost in terms of total healthcare cost from polyclinic, emergency department, hospital admissions and SOC visits from one year preceding the first annual review to the period between the first and the second annual review

### Other pre-specified outcomes

Additionally, there are other planned outcomes to be evaluated.

#### Clinical and Biochemical Indicators

- Change in mean body weight (kg) of patients
- Change in proportion of patients meeting their target blood pressure levels
- Change in proportion of patients meeting their target LDL-cholesterol levels

#### Lifestyle Measures

- Change in exercise duration (minutes per week) in patients
- Change in smoking prevalence and cigarettes smoked daily in patients
- Change in proportion of patients with community resource utilisation in the past year

#### Screening Adherence

- Change in diabetes retinal photography rates, diabetes foot screening rates in patients

## Sample size calculation

The sample size calculation was based on HbA1c, the primary outcome measure, for which a difference of 0.5 percentage points was taken to be clinically meaningful. The sample size of 1620 was calculated based on 1:1 allocation into the Intervention and Control, 6 pre-specified subgroups (with 3 age tertiles and 2 gender groups), at a power of 90%, with an alpha value of 0.05 (95% confidence interval) and accounting for an attrition rate of 30%. The study team aims to recruit a total of 1620 patients across the four sites.

## Statistical methods

### Statistical analyses

Descriptive statistics of the baseline data will be reported, and exploratory data analysis will be conducted prior to model building. Continuous variables will first be tested for normality of distribution. Bivariate analyses will be conducted for continuous variables, and chi-squared test or Fisher’s exact test for categorical variables will be used to determine statistically significant differences between the Intervention and the Control for each baseline characteristic. The analysis will be conducted with the intention-to-treat (ITT) principle unless otherwise specified. Missing endpoints will be imputed with the last observation carried forward (LOCF).

Generalised linear regression will be used to analyse the study endpoint (HbA1c) and while controlling for baseline HbA1c as a covariate. PAM-13 score and other secondary continuous outcomes variables will follow the same analysis methods unless otherwise stated. Logistic regression will be utilised to assess binary outcomes such as screening compliance in the past year, smoking status, and frequent exercise status. Covariates and interaction terms to be included in the model will be selected through inquiry of the data and reviewing existing literature. A stepwise process will be used to drop covariates that have a *p*-value greater than or equal to 0.2. Akaike information criterion (AIC) will be used to decide on the linear model used for potentially non-normal dependent variables such as total cost. Covariates included in the various models will be reported. The outcomes will be compared between the Intervention and the Control. Statistical tests will be conducted at a 2-sided alpha level of 0.05 with confidence intervals calculated at 95%, 2-sided unless otherwise stated. All analysis will be conducted using the STATA software version 16.

### Subgroup analyses

HbA1c levels are known to be related to gender and age amongst patients with diabetes in various population groups, which may be attributed to a multitude of factors such as biological differences and disease progression status. However, the relationship between gender and patient activation has yielded inconsistent results in various different population context [30, 31]. To elucidate these relationships under the unique context, pre-specified subgroup analysis will be conducted to evaluate HbA1c. The model for subgroup analysis would include treatment group, time, with sex and age category (3 strata, “18–49”, “50–64” & “65 and above”) as fixed effects, adjusting for covariates identified in model building. The significance will be adjusted using false discovery rate correction to account for multiple testing [32].

Exploratory analysis will also be conducted for the variables HbA1c and PAM-13 score to examine the effects of the programme across different population subgroups. The following subgroups will be assessed: ethnicity, years since diagnosis of diabetes at baseline, HbA1c at baseline, education, and baseline insulin use. The purpose of the analyses is for exploratory purposes and to guide hypothesis generation and inform subsequent research studies [33].

### Healthcare cost and utilisation

Healthcare cost consists of the total cost incurred for providing care. It includes the relevant labour, medication, room, service and administrative costs prior to any form of subsidies or reimbursement. The aggregate cost and utilisation 1 year prior to recruitment will be taken as the baseline and compared with the aggregate cost and utilisation between the first to the second annual review.

Total healthcare cost will be examined for both polyclinic and overall hospital care utilisation from relevant EMRs or billing record systems. Cost data will be standardised to a full year for fair comparison. Change in healthcare cost between Intervention and Control will be modelled using generalised linear regression with log-link and gamma distribution or difference-in-difference analysis with covariate adjustment. Healthcare utilisation (polyclinic attendance, SOC, emergency departments, hospital admissions), which are positive count data, will be modelled using Poisson or zero inflated negative binomial regression adjusting for covariates following preliminary analysis [34].

## Dissemination policy

Findings from the study will be disseminated through presentations at international conferences, publications in medical journals or other media platforms. They will also be communicated to the Singapore Ministry of Health and other key stakeholders of the project.

## Discussion

PACE-D evaluates the effectiveness of personalised CSP in persons living with diabetes. The multi-pronged programme aims to help patients prepare for their consultation, to participate collaboratively in discussions about their goals and how they might pursue them, and to facilitate their participation in community programmes that support self-management. The interventions in the programme leverage the strengths of the existing team-based care model adopted in the polyclinics that is intended to allow better relationship to be built between patients and their care team, to improve patient experience, and possibly to improve clinical outcomes. In addition, this trial is unique in that the CSPs are practised in the multicultural and multilingual Singapore primary care setting – CSPs are conducted in the English, Malay and/or Chinese language depending on the language proficiencies of the enrolled patients and the CSP practitioners.

In designing and implementing this complex trial which entails measuring and evaluating outcomes across the intervention and control groups over pre- and post- interventions, a number of challenges in wide-ranging areas have been encountered. They include the transfer of knowledge and skills of the UK YoC Partnerships care model to a local core team of doctors and nurses accredited as qualified local trainers in Singapore; adapting the training materials to the local primary care context by the accredited local trainers who went on to conduct training for the CSP practitioners; developing technical and computer skills to operationalise the preparation of CPLs; and integrating CSP conversations as part of routine care that require additional consultation time from the CSP practitioners.

While the one-two year timeline may be adequate to see initial improvements in clinical outcomes, the duration may not be long enough for the full effects of the CSP model of care to be observed or understood. Nonetheless, the key strength of PACE-D is that this is a pragmatic trial conducted in routine primary care practices where HCPs have to grapple with a scarcity of time and resources to deliver holistic care to patients with LTCs. Successful implementation of the programme therefore affirms the new care model is feasible in real world non-study settings and supports greater generalisability of the study findings for other primary care practices. This study will increase understanding of the role of personalised CSPs in the delivery of care for persons living with diabetes.

## Supplementary information

**Additional file 1.**

## Data Availability

Not applicable.

## Abbreviations

AR: Annual Review
BI: Business Intelligence
CC: Care Coordinator
CM: Care Manager
CSP: Care and Support Planning
CPL: Care Planning Letter
ePOS: Electronic Polyclinic Outpatient System
EMR: Electronic Medical Record
HbA1c: Glycated Haemoglobin
HCP: Health Care Provider
ITT: Intention-to-treat
LOCF: Last Observation Carried Forward
LTC: Long Term Condition
MOH: Ministry of Health
NUP: National University Polyclinics
NUHS: National University Health System
PACE-D: Patient Activation through Community Empowerment/Engagement for Diabetes Management
PAM-13: Patient Activation Measure-13
PC: PACE-D Coordinator
PCP: Primary Care Physician
SGD: Singapore Dollar
SOC: Specialist Outpatient Clinic
UK: United Kingdom
YoC: Year of Care
