# Supplementary material for: The Patient Activation through Community Empowerment/Engagement for Diabetes Management (PACE-D) protocol: a non-randomised controlled trial of personalised care and support planning for persons living with diabetes
Source: BMC Fam Pract. 2020 Jun 19;21:114. doi: 10.1186/s12875-020-01173-2 (PMC7305581; doi:10.1186/s12875-020-01173-2)
Supplement: Supplementary file 1 — Additional file 1. [file 12875_2020_1173_MOESM1_ESM.pdf]

Annex A: Care Planning Letter

Persons enrolled on PACE-D receive the care planning letter (CPL) two weeks before their upcoming care and support planning (CSP) conversation. The CPL comprises eight user-friendly pages that provide information on the status of their latest investigations and biomarkers, with space for patients to jot down notes on their thoughts, questions, as well as goals that may be discussed at the CSP conversation. See excerpts in Supplementary Figures 1 and 2.

Supplementary Figure 1. Excerpt of the care planning letter to prompt agenda setting in preparation for the upcoming CSP conversation

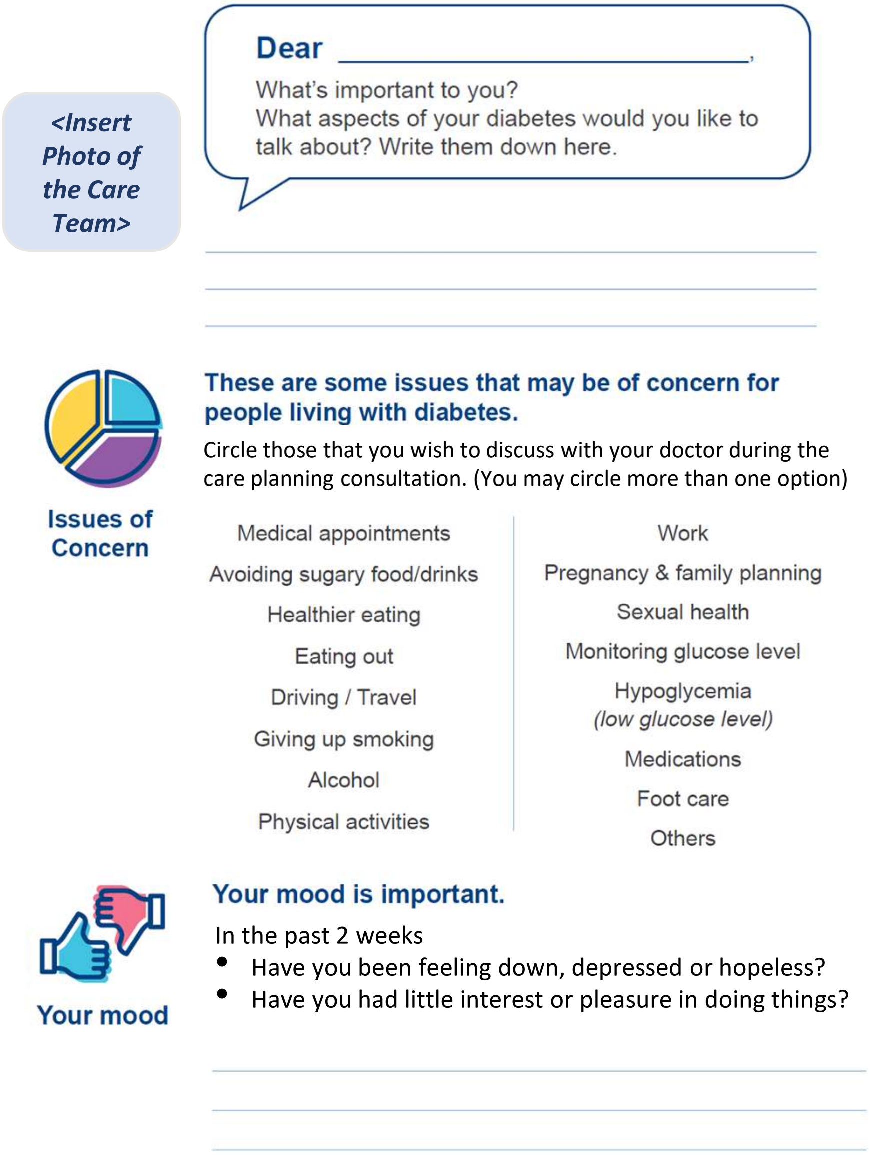

<Insert Photo of the Care Team>

Dear ____________________________,

What's important to you?
What aspects of your diabetes would you like to talk about? Write them down here.

Issues of Concern

These are some issues that may be of concern for people living with diabetes.

Circle those that you wish to discuss with your doctor during the care planning consultation. (You may circle more than one option)

Medical appointments	Work
Avoiding sugary food/drinks	Pregnancy & family planning
Healthier eating	Sexual health
Eating out	Monitoring glucose level
Driving / Travel	Hypoglycemia *(low glucose level)*
Giving up smoking	Medications
Alcohol	Foot care
Physical activities	Others

Your mood

Your mood is important.

In the past 2 weeks

- Have you been feeling down, depressed or hopeless?
- Have you had little interest or pleasure in doing things?

Supplementary Figure 2. Excerpt of the care planning letter detailing the HbA1c and LDL-cholesterol results

Blood Glucose Control (HbA1c)

HbA1c tells you how your blood glucose has been over the last 8 to 12 weeks. Keeping your HbA1c on target will lower your risk of diabetes complications like blindness and kidney failure.

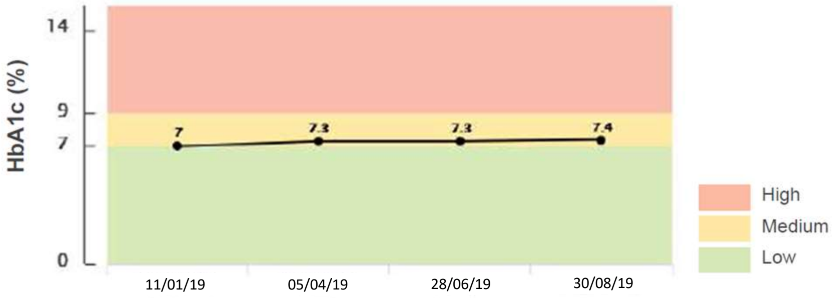

Your questions or thoughts:

Your risk:
Medium

LDL Cholesterol

This is a measure of bad cholesterol in the blood. Maintaining a good level will reduce your risk of heart attack and stroke.

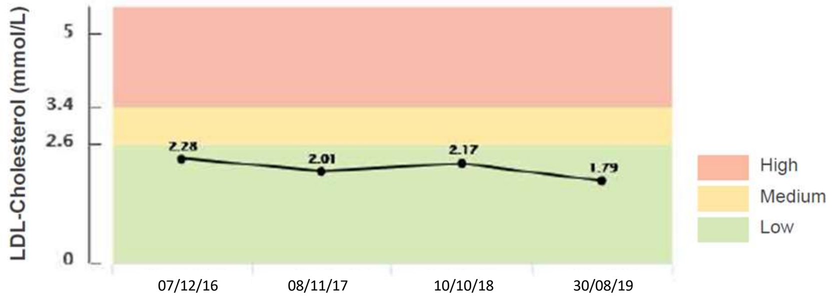

Your questions or thoughts:

Your risk:
Low
